# Supplementary material for: Rebound of shelf water salinity in the Ross Sea
Source: Nat Commun. 2019 Nov 29;10:5441. doi: 10.1038/s41467-019-13083-8 (PMC6884573; doi:10.1038/s41467-019-13083-8)
Supplement: Supplementary file 1 — Supplementary Information [file 41467_2019_13083_MOESM1_ESM.pdf]

Supplementary Information

“Rebound of shelf water salinity in the Ross Sea”

By P. Castagno et al.

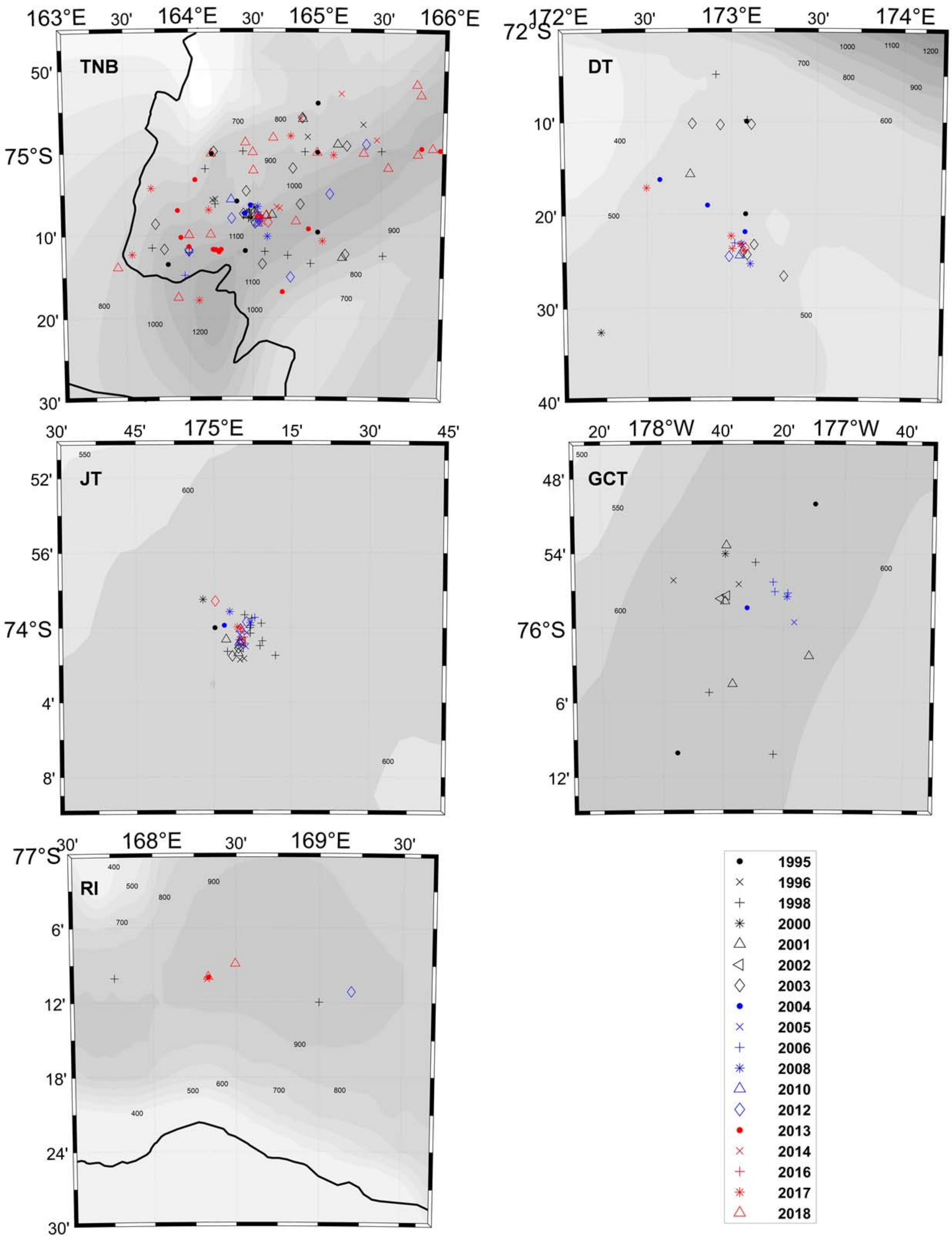

Supplementary Figure 1. Location of the CTD casts used for each study area: Terra Nova Bay (TNB); Drygalski Trough mouth (DT); Ross Island depression (RI); Joides Trough (JT); and Glomar Challenger Trough (GCT). The bold line in TNB and RI is the ice outline.

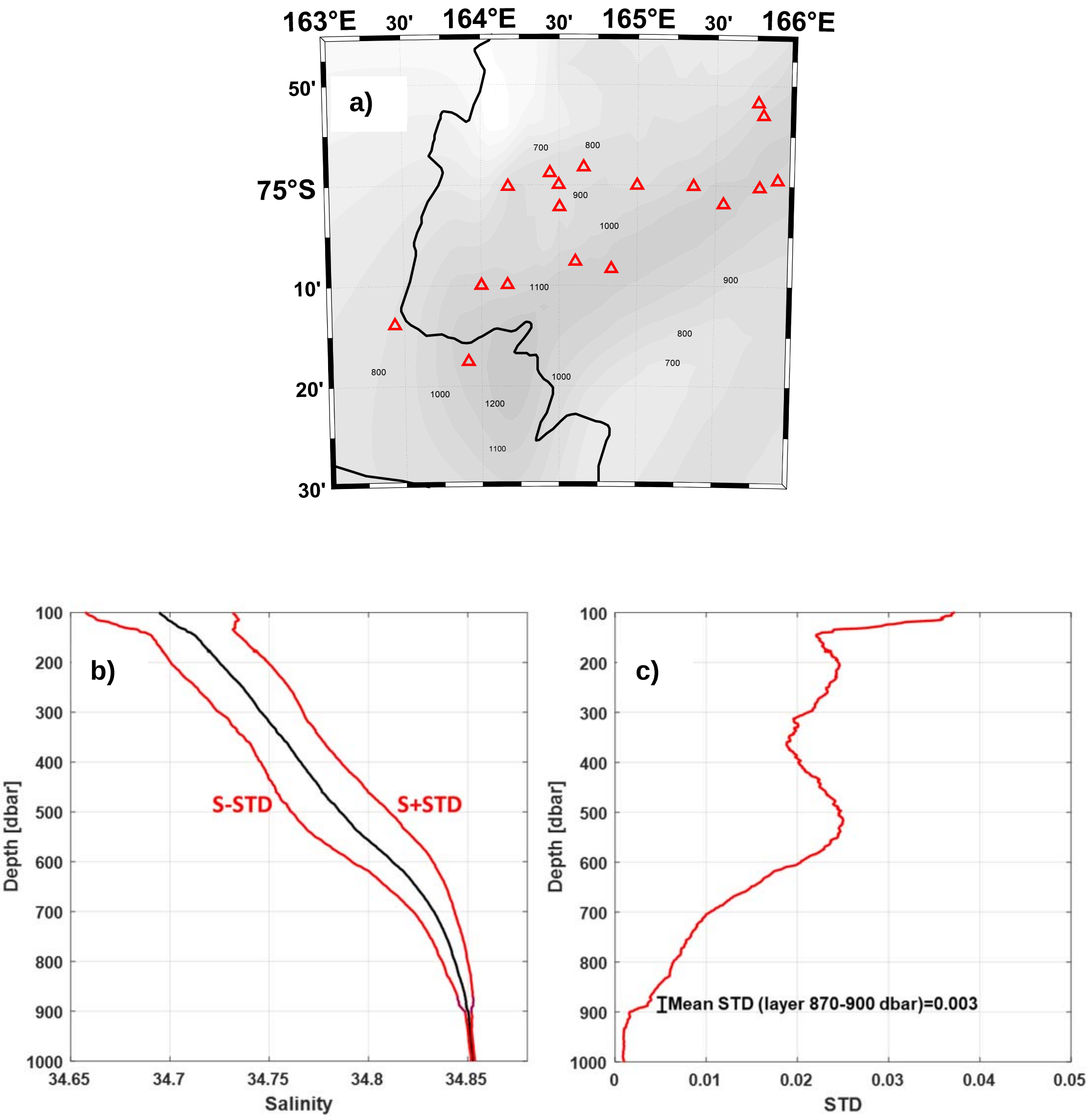

Supplementary Figure 2. a) Map of the CTD casts in the TNB region in 2018, b) mean salinity profile (black line) of the 2018 casts with ± the one standard deviation (STD, red lines) evaluated on pressure surfaces, c) standard deviation (red line) profile obtained for each pressure surface of 1 dbar.

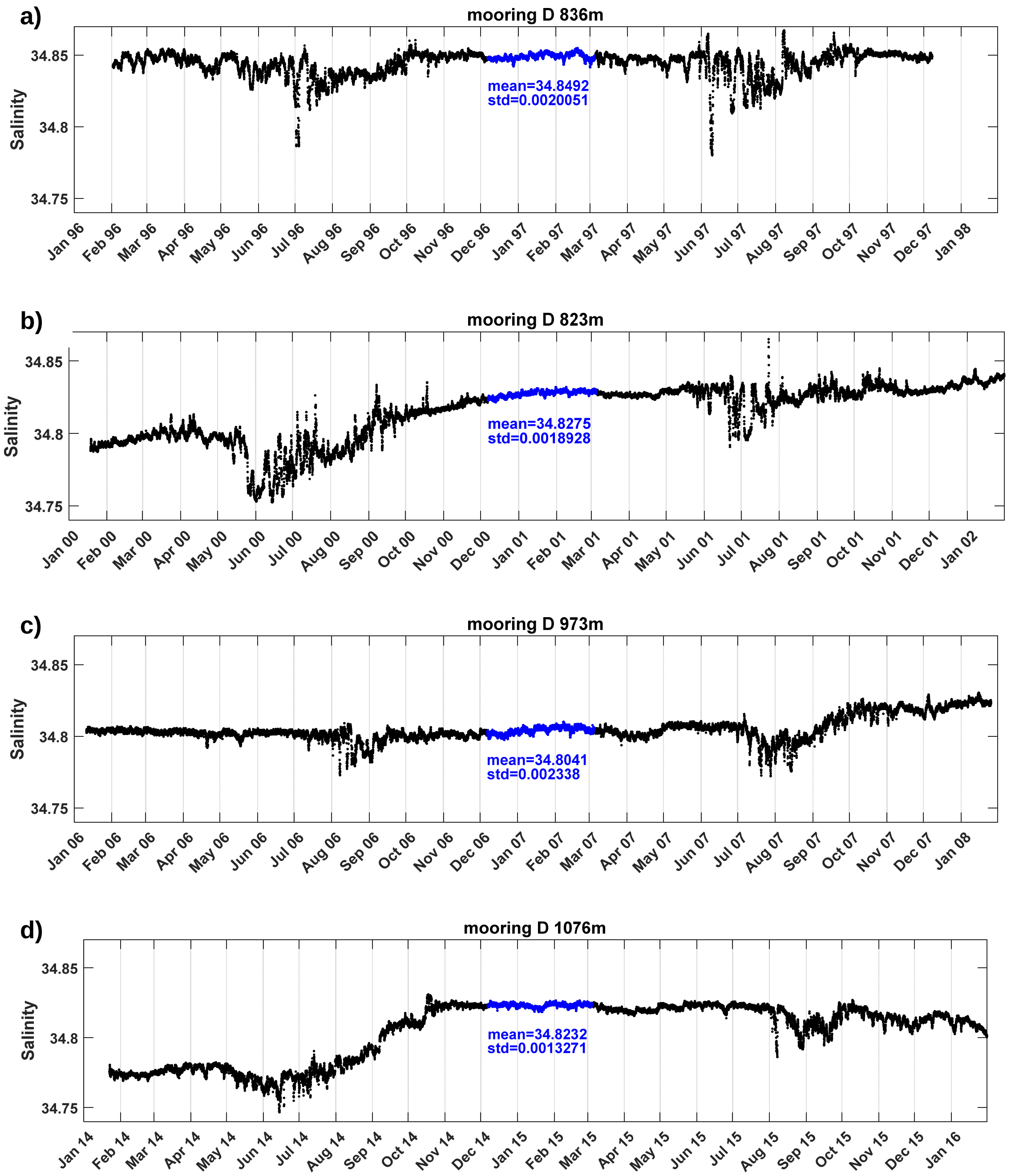

Supplementary Figure 3. Time series of HSSW salinity in TNB. Near-bottom salinity measured at mooring D of the MORSea project in the centre of TNB (75.14°S, 164.55°E) at depths of a) 836m from February 1996 to December 1997, b) 823 m from January 2000 to February 2002, c) 973m from January 2006 to January 2008, and d) 1076 m from January 2014 to February 2016.

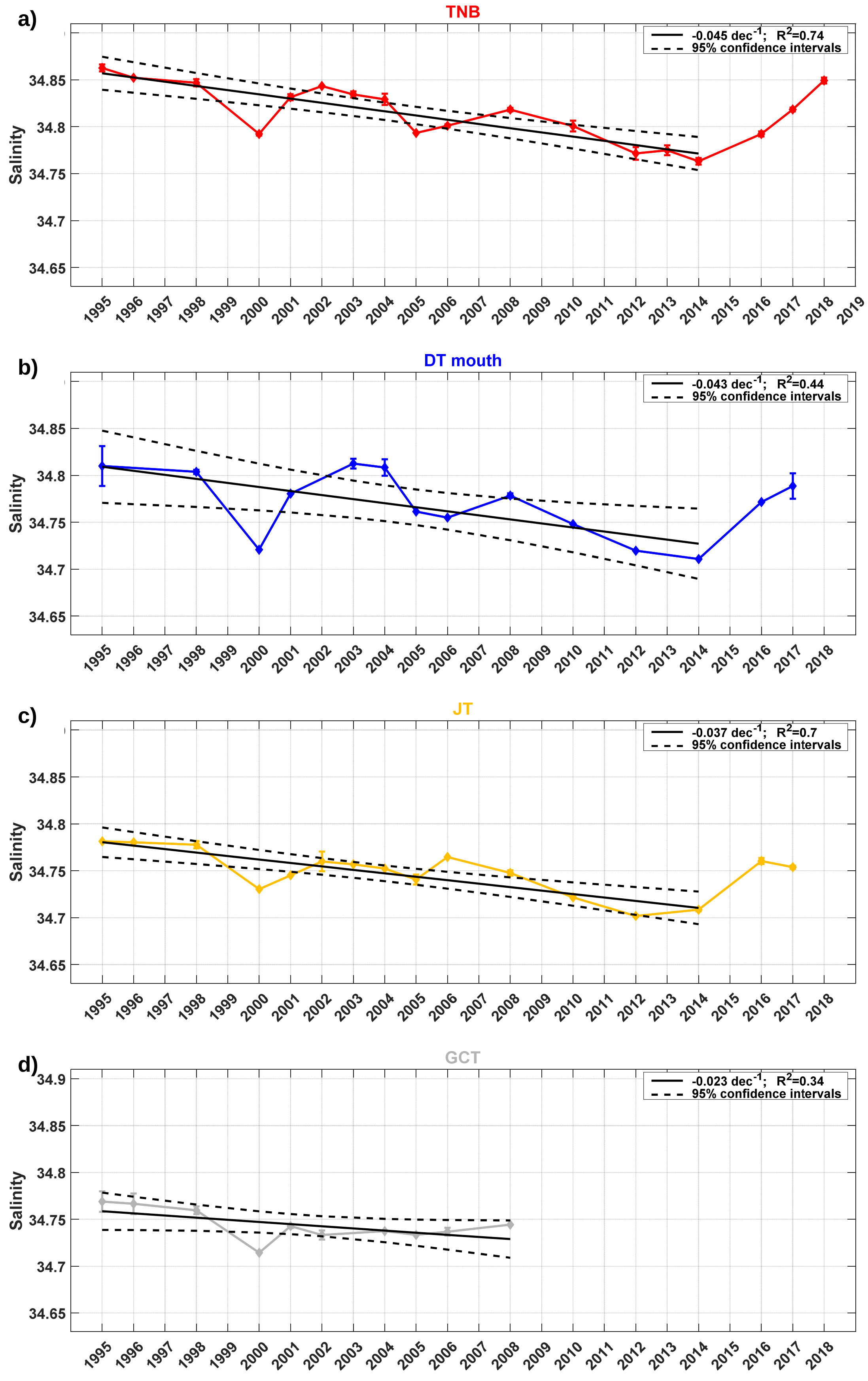

a)
TNB
-0.045 dec-1; R2=0.74
95% confidence intervals
Salinity
34.85
34.8
34.75
34.7
34.65
1995 1996 1997 1998 1999 2000 2001 2002 2003 2004 2005 2006 2007 2008 2009 2010 2011 2012 2013 2014 2015 2016 2017 2018 2019
b)
DT mouth
-0.043 dec-1; R2=0.44
95% confidence intervals
Salinity
34.85
34.8
34.75
34.7
34.65
1995 1996 1997 1998 1999 2000 2001 2002 2003 2004 2005 2006 2007 2008 2009 2010 2011 2012 2013 2014 2015 2016 2017 2018
c)
JT
-0.037 dec-1; R2=0.7
95% confidence intervals
Salinity
34.85
34.8
34.75
34.7
34.65
1995 1996 1997 1998 1999 2000 2001 2002 2003 2004 2005 2006 2007 2008 2009 2010 2011 2012 2013 2014 2015 2016 2017 2018
d)
GCT
-0.023 dec-1; R2=0.34
95% confidence intervals
Salinity
34.9
34.85
34.8
34.75
34.7
34.65
1995 1996 1997 1998 1999 2000 2001 2002 2003 2004 2005 2006 2007 2008 2009 2010 2011 2012 2013 2014 2015 2016 2017 2018

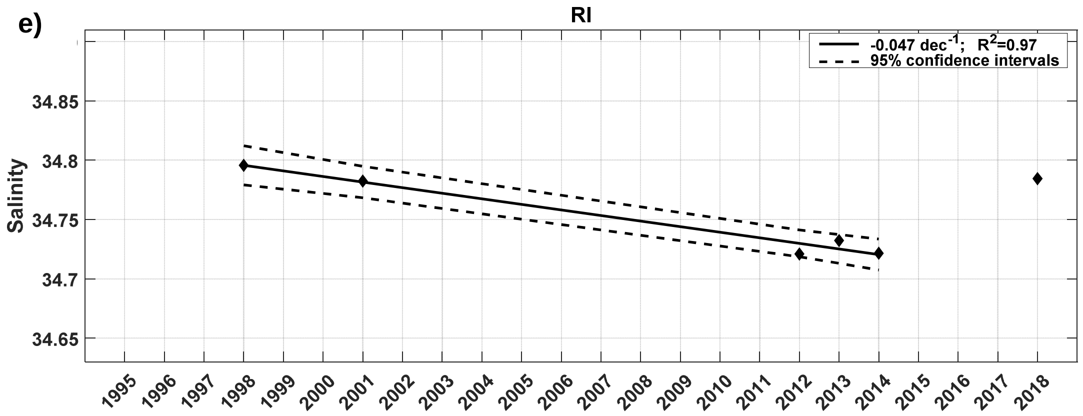

Supplementary Figure 4. Salinity time series from Fig. 2 with linear trends. Trend lines show regressions of salinity change per decade from 1995 to 2014, with the coefficient of determination and the 95% confidence interval, for a) TNB (trend significant at 99% level), b) DT mouth (trend significant at 98% level), c) JT (trend significant at 99% level), d) GCT (trend not statistically significant), and e) RI (trend not statistically significant).

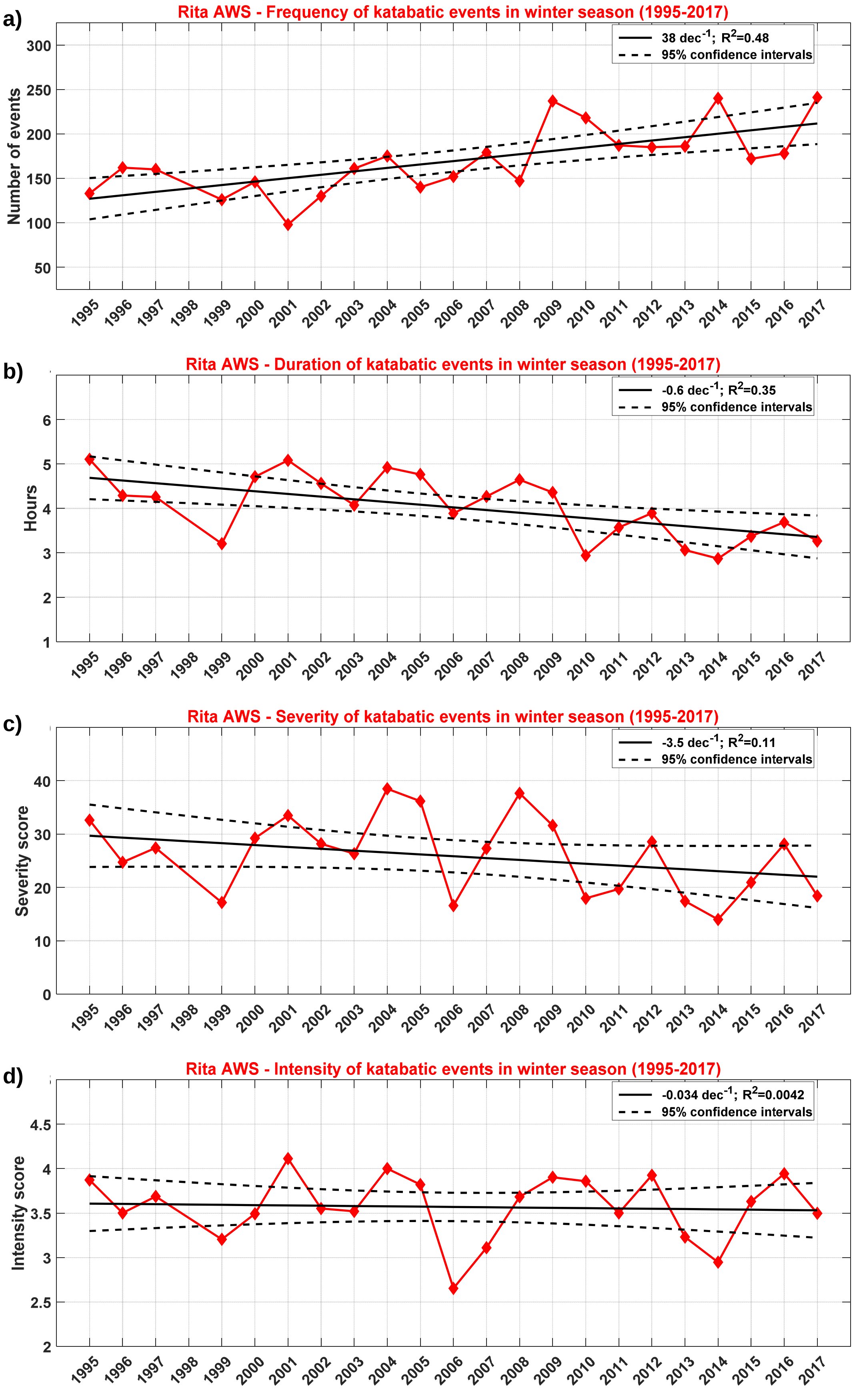

a)
Rita AWS - Frequency of katabatic events in winter season (1995-2017)
38 dec^{-1}; R^2=0.48
95% confidence intervals
Number of events
b)
Rita AWS - Duration of katabatic events in winter season (1995-2017)
-0.6 dec^{-1}; R^2=0.35
95% confidence intervals
Hours
c)
Rita AWS - Severity of katabatic events in winter season (1995-2017)
-3.5 dec^{-1}; R^2=0.11
95% confidence intervals
Severity score
d)
Rita AWS - Intensity of katabatic events in winter season (1995-2017)
-0.034 dec^{-1}; R^2=0.0042
95% confidence intervals
Intensity score

Supplementary Figure 5. Changes in katabatic winds at TNB, 1995 to 2017. Time series with linear regressions of change per decade from 1995 to 2017, coefficient of determination and the 95% confidence interval for a) katabatic events frequency, (trend significant at 99% level), b) duration (trend significant at 99% level), c) severity (trend not statistically significant), and d) intensity (trend not statistically significant). The time series have been obtained by analysing the wind data collected by Rita AWS during the winter season (April to October).

Supplementary Tables

Study area	Year	Number of profiles averaged	First CTD profile	Last CTD profile
TNB	1995	7	19/02/1995	20/02/1995
TNB	1996	4	14/01/1996	05/02/1996
TNB	1998	13	07/12/1997	22/02/1998
TNB	2000	2	16/01/2000	18/01/2000
TNB	2001	9	17/01/2001	19/01/2001
TNB	2002	2	31/01/2002	03/02/2002
TNB	2003	13	17/01/2003	27/01/2003
TNB	2004	2	05/01/2004	27/01/2004
TNB	2005	1	15/01/2005	
TNB	2006	4	08/01/2006	16/01/2006
TNB	2008	3	25/01/2008	01/02/2008
TNB	2010	2	03/02/2010	06/02/2010
TNB	2012	8	23/01/2012	09/02/2012
TNB	2013	18	14/02/2013	05/03/2013
TNB	2014	6	11/01/2014	27/01/2014
TNB	2016	2	31/01/2016	06/02/2016
TNB	2017	9	08/01/2017	11/02/2017
TNB	2018	19	09/01/2018	17/02/2018
DT	1995	2	12/02/1995	12/02/1995
DT	1998	2	26/12/1997	27/12/1997
DT	2000	1	27/01/2000	
DT	2001	1	28/01/2001	
DT	2003	7	06/02/2003	07/02/2003
DT	2004	3	25/02/2004	26/02/2004
DT	2005	1	19/01/2005	
DT	2006	1	29/01/2006	
DT	2008	2	27/01/2008	04/02/2008
DT	2010	1	30/01/2010	
DT	2012	1	26/01/2012	
DT	2014	1	22/01/2014	

DT	2016	1	28/01/2016	
DT	2017	4	04/02/2017	09/02/2017
JT	1995	1	15/02/1995	
JT	1996	2	13/01/1996	26/01/1996
JT	1998	10	16/12/1997	11/02/1998
JT	2000	1	15/01/2000	
JT	2001	2	20/01/2001	21/01/2001
JT	2002	2	30/01/2002	07/02/2002
JT	2003	3	05/02/2003	05/02/2003
JT	2004	1	13/01/2004	
JT	2005	3	29/01/2005	02/02/2005
JT	2006	2	27/01/2006	28/01/2006
JT	2008	2	27/01/2008	03/02/2008
JT	2010	2	31/01/2010	11/02/2010
JT	2012	2	27/01/2012	28/01/2008
JT	2014	2	06/01/2014	30/01/2014
JT	2016	2	24/01/2016	27/01/2016
JT	2017	2	17/01/2017	30/01/2017
GCT	1995	4	01/02/1995	02/02/1995
GCT	1996	2	23/01/1996	23/01/1996
GCT	1998	3	13/12/1997	13/12/1997
GCT	2000	1	22/01/2000	
GCT	2001	4	11/02/2001	12/02/2001
GCT	2002	2	21/01/2002	29/01/2002
GCT	2004	1	08/01/2004	
GCT	2005	1	31/01/2005	
GCT	2006	3	21/01/2006	21/01/2006
GCT	2008	1	28/01/2008	
RI	1998	2	29/01/1998	29/01/1998
RI	2001	1	31/01/2001	
RI	2012	1	05/02/2012	
RI	2013	1	12/02/2013	
RI	2014	1	01/02/2014	
RI	2018	2	23/01/2018	23/01/2018

Supplementary Table 1. Summary of the number and date of CTD profiles used to construct the austral summer average in reach region.

Supplementary Discussion

In order to evaluate the impact of katabatic wind forcing in TNB on the variability of HSSW salinity, we analysed wind speed and direction recorded by Automatic Weather Station (AWS) Rita between 1995 and 2017, with a time resolution of one hour.

Rita is part of the PNRA meteo-climatological observatory and is considered the most representative AWS site of the atmospheric conditions upstream of Terra Nova Bay polynya[1].

To identify a katabatic event, we defined an objective criterion based on the analysis of the wind rose diagram and of wind class frequency distribution: an event occurs when the wind speed is equal or greater than the 90th percentile level for at least one hour (for the investigated study period, 22.1 m s^{-1}) and the wind direction is between 225° and 360°. We considered only katabatic events in winter (April to October), when HSSW is produced. The katabatic wind events were characterized by frequency (number of katabatic events per year), average duration, severity (sum of the differences between the measured wind speed and the relative percentile level) and intensity (ratio between severity and duration). Thus, the severity score highlights the magnitude of a katabatic event, measured in terms of deviation from the reference percentile level, while the intensity score provides information about variability and trends of strong and short events.

We found no significant correlation between the time series of HSSW salinity (Fig. 2) and the four katabatic wind descriptors (Fig. S5). Furthermore, we observe an opposite behavior of the frequency and duration time series. The first exhibit a positive trend (+38 events dec^{-1}, significant at 95% level), whereas, the duration shows a negative trend (-0.7 h dec^{-1}, significant at 95% level). Moreover, in the katabatic wind descriptors we do not detect the same fluctuation of 5-10 years observed in the TNB salinity time series.

These results suggest that the salinity trends and fluctuations of the HSSW measured in TNB from 1995 to 2018 do not depend on the katabatic wind regime in TNB.

Supplementary References

1. Knuth, S. L. & Cassano, J. J. An analysis of near-surface winds, air temperature, and cyclone activity in Terra Nova Bay, Antarctica, from 1993 to 2009. *J. Appl. Meteorol. Climatol.* **50**, 662–680 (2011).
